# Accurate contrast determination for X-ray speckle visibility spectroscopy

**DOI:** 10.1107/S1600577520006773

**Published:** 2020-06-19

**Authors:** Yanwen Sun, Jordi Montana-Lopez, Paul Fuoss, Mark Sutton, Diling Zhu

**Affiliations:** a Linac Coherent Light Source, SLAC National Accelerator Laboratory, USA; b Physics Department, Stanford University, USA; c Physics Department, McGill University, USA

**Keywords:** X-ray speckle visibility spectroscopy, XPCS, photon locating algorithms

## Abstract

X-ray speckle visibility spectroscopy using X-ray free-electron lasers has long been proposed as a probe of fast dynamics in noncrystalline materials. In this paper, numerical modeling is presented to show how the data interpretation of visibility spectroscopy can be impacted by the nonidealities of real-life X-ray detectors. Using simulated detector data, this work provides a detailed analysis of the systematic errors of several contrast extraction algorithms in the context of low-count-rate X-ray speckle visibility spectroscopy and their origins are discussed. Here, it was found that the finite detector charge cloud and pixel size lead to an unavoidable ‘degeneracy’ in photon position determination, and that the contrasts extracted using different algorithms can all be corrected by a simple linear model. The results suggest that experimental calibration of the correction coefficient at the count rate of interest is possible and essential. This allows computationally lightweight algorithms to be implemented for on-the-fly analysis.

## Abstract 2

A numerical model that can mimic closely the behavior of real X-ray detectors in the context of X-ray speckle visibility spectroscopy is described. Using this model, this work investigates the origin of discrepancies between commonly used photon locating algorithms and proposes a calibration routine to resolve the discrepancy.

## 1. Introduction   

X-ray photon correlation spectroscopy (XPCS) studies the dynamics of amorphous and disordered material systems via measuring the fluctuations of speckle patterns resulting from coherent X-ray scattering (Grübel *et al.*, 2008 ▸). Dynamics is revealed through the time-dependent intermediate scattering function extracted by calculating the intensity autocorrelations between speckle patterns recorded in sequence. Alternatively, X-ray speckle visibility spectroscopy enables access to the same information at faster time scales than the frame rate of the detectors by examining the speckle visibility as a function of detector exposure time (Inoue *et al.*, 2012 ▸; DeCaro *et al.*, 2013 ▸; Li *et al.*, 2014 ▸; Verwohlt *et al.*, 2018 ▸; Möller *et al.*, 2019 ▸). The advent of X-ray free-electron lasers (FELs) opens up the possibility for capturing ultrafast atomic-scale dynamics with speckle visibility measurement by using a pair of femtosecond X-ray pulses with time separation ranging from femtoseconds to nano­seconds to define the ‘exposure time’ (Gutt *et al.*, 2009 ▸; Stephenson *et al.*, 2009 ▸; Emma *et al.*, 2010 ▸; Altarelli, 2011 ▸; Ishikawa *et al.*, 2012 ▸; Kang *et al.*, 2017 ▸; Milne *et al.*, 2017 ▸).

In order to probe the dynamics of interest without perturbing the system with the X-ray pulse itself, the single-pulse radiation dose must be limited. This leads to one of the main challenges for speckle visibility measurements at X-ray FELs: the very low intensity of the speckle patterns (Hruszkewycz *et al.*, 2012 ▸; Perakis *et al.*, 2018 ▸; Roseker *et al.*, 2018 ▸). In this limit, contrast cannot be easily determined via direct autocorrelation calculations. Photon statistics analysis see Appendix *A* [App appa]) was found to be an effective alternative to estimate the speckle contrast, *i.e.* via quantifying the observed probabilities of multiple photons per pixel events using pixelated detectors with single-photon sensitivity. Spatial resolution of pixelated detectors can be compromised for hard X-rays due to charge sharing between neighboring pixels (Dufresne *et al.*, 1995 ▸) which complicates the process of assignment of photons to individual pixels. Hruszkewycz *et al.* (2012 ▸) demonstrated that it is possible to overcome this effect via a droplet algorithm (Livet *et al.*, 2000 ▸) based analysis protocol and determine the locations of each detected photon for speckle contrast evaluation. Other experiments (Yoon, 2016 ▸; Perakis *et al.*, 2018 ▸) adopted a faster algorithm that skips the step of ‘dropletizing’ and counts the photon numbers of each pixel directly from its readout based on a simpler model of charge cloud in order to extract visibility from speckle patterns. However, we discovered that these two contrast extraction algorithms returned significantly different contrast values from the same experimental dataset, prompting further investigation and comparison between popular photon assignment and speckle contrast extraction methods.

In this work, we present a systematic analysis of the accuracy of speckle visibility extraction by most frequently used algorithms in the low photon count rate regime. The impact of charge cloud sharing between pixels, pixel readout noise, gain non-uniformity, among other nonidealities of current hard X-ray detectors are considered. We show via simulation that, although the contrast extraction algorithms rarely give the correct ‘absolute’ contrast value, they can all be calibrated using a simple linear model. Varying the detector simulation parameters indicates that the procedure is generally applicable. Following the proposed calibration routine, computationally light-weight algorithms can be used to return sufficiently accurate and near-real-time analysis during the experiment to provide crucial live feedback.

## 2. Detector simulation   

**Figure 1:**
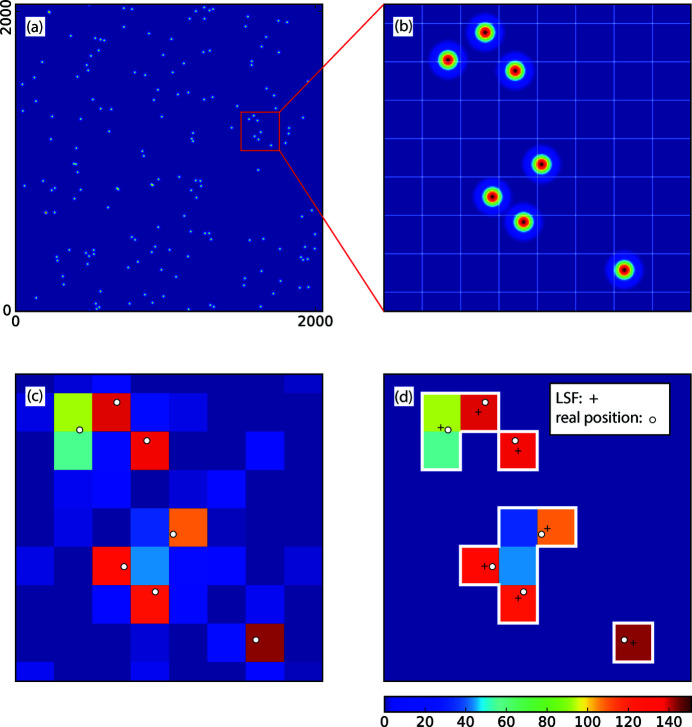
Charge sharing in pixelated X-ray detectors and photon location recovery. (*a*) Simulated sparse Gaussian charge clouds generated from weak X-ray scattering. (*b*) Magnified view of a region of interest from (*a*), showing an 8 × 8 pixel area. The solid white lines indicate the pixel boundaries. One can see photons that straddle across pixel boundaries. (*c*) Simulated detector image by binning (*b*) down to the outlined pixel grid and adding readout noise. Centers of the charge cloud representing the photon locations are indicated by circles. (*d*) Detector image after setting pixel readouts below a threshold to 0. In total four droplets are identified as indicated by the boundaries drawn in white. Photon positions recovered by the algorithm used in the work by Hruszkewycz *et al.* (2012 ▸) are plotted with ‘+’. The color bar is shared by (*c*) and (*d*). The nominal one photon readout is 151 in our simulation.

In this section, we describe the numerical model we used to simulate the detector response to X-ray photons and discuss the process of photon position determination. We first generated speckle patterns with well defined speckle size and visibility. Many independent speckle patterns were simulated by adding random phases to point scatterers, followed by a fast Fourier transform corresponding to the X-ray scattering process. The speckle size is controlled by the size of the region where scatterers are placed, effectively adjusting the oversampling ratio. Well defined visibility is achieved by summing up different numbers of independent speckle patterns (Goodman, 2007 ▸). Positions of photons, or photon maps, were then created to replicate an average count rate at the sparse photon limit (≪1 photon per pixel) using the intensity variation of the speckle pattern as the probability density of the photon events following Poisson statistics. These can be seen as the bright spots in Fig. 1 ▸(*a*) which are the results of individual photoionization events that took place in the sensor.

Each photoionization event inside the sensor material creates a charge cloud that is then collected by the electrodes of individual pixels. In a typical hard X-ray detector used for XPCS measurements, the charge cloud size is similar to the pixel size. As a result, frequently, when the photoionization takes place near the pixel boundaries, the charges generated will be split up and collected by a few neighboring pixels. This is the so-called charge-sharing effect. As photons can arrive at any position within a detector pixel, the photon map was initially generated based on a larger and much more finely sampled speckle pattern, 2048 × 2048 in our case. Charge sharing was then modeled with the convolutions of the photon maps with kernels of different shapes, *e.g.* Gaussian, Lorentzian, Super-Gaussian, *etc*. with *S* being the FWHM of the shape. Sub-pixel image shift with interpolation was used to further reduce the impact of the ‘digitization’ of the charge cloud before assigning the charges to the pixel level. We next divided up the charges into a lower resolution grid (64 × 64) that represents the pixels as shown in Fig. 1 ▸(*b*).

After distributing the X-ray generated charges to the pixel grid, the gain inhomogeneity and readout noise are introduced. The inhomogeneity of the detector in terms of the pixel-to-pixel gain variation is modeled by multiplying the detector image with a gain map which has a variation defined as a Gaussian distribution centered around 1 and with σ_g_ as its standard deviation. Electronic readout noise is introduced pixel-wise following also a Gaussian distribution with an average value 0 and a tunable standard deviation σ_r_. A typical simulated pixelated signal map is shown in Fig. 1 ▸(*c*), together with the real positions of photons denoted by the black-edged circles. The github repository for generating the detector images is located at https://github.com/Yanwen-Sun/detectorSimulation.

The algorithms for tracing back where the photons landed on the detector consist of two main steps. The first step is called ‘dropletizing’ which identifies connected pixels with signals above the detector noise, as illustrated in Fig. 1 ▸(*d*) outlined by white boundaries. The second step is called ‘photon assignment’ where, for example, a fitting algorithm is used to estimate photon positions, which are denoted with a ‘+’ sign in Fig. 1 ▸(*d*). One usually sees small discrepancies between the real and fitted photon positions. This in turn can impact the speckle visibility analysis in experiments.

**Figure 2:**
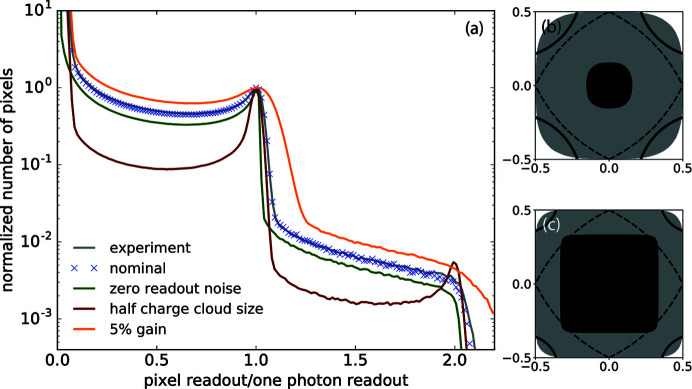
Pixel histogram of ePix100 data (gray) at 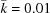 photons per pixel. (*a*) Blue crosses, green, red and orange lines are histograms from the detector simulations with different parameters. The histograms are normalized to the first photon peak for easier comparison. Panels (*b*) and (*c*) map out regions within a single pixel that have different single-photon contribution fractions. The black area denotes the region where a photon will contribute 98% of its generated charges to the readout of this pixel. The gray area denotes the region for more than 50% of the charges to be collected by this pixel. The dashed black lines are the 50% charge contribution boundary for the LSF model. The corners defined by the solid black lines are the areas where one photon contributes at least 2% (3 out of 151 ADUs) of the total charges to each of its three neighboring pixels. Panel (*b*) assumes a charge cloud size of *S* = 0.385 (units in pixel size). The percentages of the black, gray, white and corner areas are namely 8.3%, 82.3% and 10.4% and 21.4%, respectively. Panel (*c*) assumes *S* = 0.193, or a factor of two smaller than (*b*). The percentages of the black, gray, white and corner areas are 43.6%, 97.5%, 2.5% and 5.3%, respectively.

Following the process discussed above, we were able to replicate the general behavior of the ePix100 (Sikorski *et al.*, 2016 ▸) in terms of the analog-to-digital unit (ADU) histogram, at the mean photon density of 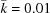 photons per pixel as shown in Fig. 2 ▸(*a*). The gray solid line is the experimental data recorded by the ePix100 detector during a speckle visibility measurement. One photon generates an equivalent of 151 ADUs. The histogram presented here is normalized by that value in the horizontal axis. A Gaussian charge cloud shape with *S* = 0.385 (in units of pixel size), σ_g_ = 0.5%, σ_r_ = 3.0 closely reproduced this histogram as indicated by the blue crosses and are defined as the nominal values for our simulation of detector images. Using the same values for these three parameters, we are also able to closely reproduce the pixel histograms at other count rates within the dynamic range of the detector. We also estimate that the variation of the charge cloud size is dominated by the diffusion of charges within the sensor resulting from different absorption depth of photons (Ren *et al.*, 2018 ▸). Given the ePix100 silicon sensor thickness of 500 µm, for 8 keV photons, the r.m.s. charge cloud size variation is 15% of the average size, producing a negligible impact to the pixel ADU histogram. The pixel readout histogram is very sensitive to the parameters chosen here. The green, red and orange curves are the simulated histograms when either *S*, σ_g_ or σ_r_ is individually changed to 0.1925, 0 and 5% while keeping the other parameters the same as the nominal values. More distinctive photon peaks generally correspond to smaller values of the three parameters.

We now look at the extent of charge sharing based on the identified simulation parameters. A charge cloud size *S* = 0.385 pixels corresponds to a 1/*e* ^2^ diameter of 2*w*_0_ = *S*(2/log2)^1/2^ ≃ 0.654. The percentage *P*_C_ of charges contained within a boundary defined by *x*_1_, *x*_2_, *y*_1_, *y*_2_ and photon location *x*_0_, *y*_0_ can be estimated as
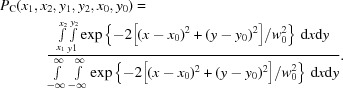


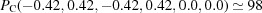
 suggests that 148 out of 151 of the pixel readout units will fall into a square of 0.84 × 0.84 pixels, which is the level of charge loss indistinguishable from the readout noise σ_r_ = 3. The boundary for one photon to contribute to more than 98% of the charge within one pixel is plotted in Fig. 2 ▸(*b*), determined by finding *x*_0_, *y*_0_ which can satisfy 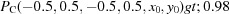. The size of the enclosed area in black is a measure of accuracy of the photon position determination within a pixel for any algorithm. One additional conclusion we can draw from the calculation is that the charges of one photon can at most be shared by 2 × 2 pixels. A further check can be obtained by looking at the single-photon droplets, as the fraction of single-pixel droplets among all one-photon droplets will agree with the fraction of the 98% region.

With the identified parameters that mimic the ePix100 detector, we next simulated weak speckle patterns with known contrasts and compare with contrast values extracted from a few commonly used photon assignment algorithms.

## 3. Contrast evaluation algorithm comparison   

Using the procedure discussed in the previous sections we were able to simulate a large quantity of detector images at different contrast and intensity levels based on the nominal parameters which replicate the ePix100 detector behavior. We chose a simulated speckle size of 2 pixels, which is defined as the FWHM of the intensity spatial correlations of the speckle pattern. This represents the typical oversampling ratio for speckle visibility spectroscopy experiments. Three commonly used algorithms are applied to correct for charge sharing and assign photons to a pixel following their respective rules. We can extract the one- and two-photon probabilities *P*(*k* = 1) and *P*(*k* = 2) given an adequate number of images, from which the contrast can be calculated through the following equation,
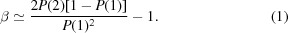
This equation can be derived by the Taylor expansion of the negative binomial distribution of *k* = 1, 2 photons per pixel with respect to 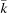 when 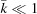. We present the detailed derivations in Appendix *A* [App appa].

The error bar for the contrast determination σ_β_ is estimated by the number of photons measured and the actual contrast β_0_, the accuracy of our measurement is thus
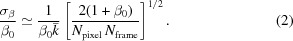
For our simulation, as mentioned in the previous section, the number of pixels for each detector image is *N*_pixel_ = 64 × 64. We chose *N*_frame_, which allows us to achieve a 10% r.m.s. precision for each contrast point, and consider this as the adequate number of images, which are plotted in Fig. 4(*a*).

We used three different photon assignment algorithms to evaluate contrast of the simulated datasets, labeled: Greedy Guess (GG), Least Squares Fit (LSF) (Hruszkewycz *et al.*, 2012 ▸), and Psana Photon Convertor (PPC) (Yoon, 2016 ▸; Thayer *et al.*, 2017 ▸); below we present a brief description of each.

*Greedy Guess*. Step 1: set all pixels below the noise threshold 5σ_r_ to 0. Identify each connected region of pixels with non-zero signal as a ‘droplet’. Step 2: identify one photon readout intensity, *r*_1_, from the droplet readout histogram and thus determine the number of photons in each droplet from its total readout. Step 3: for one-photon or one-pixel droplets, the photon position is assigned to the center of mass calculated directly from the pixel readout. Step 4: for all the other droplets: (i) if the brightest pixel has a readout value corresponding to at least one photon, assign the position of one photon to that pixel and subtract *r*_1_ from the pixel value; (ii) if the brightest pixel (*i*, *j*) has readout *r* less than *r*_1_, find its brightest neighboring pixel. A photon is assigned along the line connecting the centers of the two pixels, and the distance to pixel (*i*, *j*) is determined by (*r*_1_ − *r*)/*r*_1_. Step 5: repeat Step 4 until the photons found in Step 2 are all assigned.

*Least Squares Fit*. Step 1: use the photon assignment from GG as a starting point. Then use the calculated center of mass as the photon positions for the one-photon or one-pixel droplets. Step 2: (i) add a random number from −0.75 to 0.75 to the starting photon positions generated in GG for both the horizontal and vertical. (ii) From the starting positions, calculate the ADU values for each pixel within the droplet. The function for calculating *r*′_*i*,*j*_ [the calculated readout value of pixel (*i*,*j*)] is based on the photon positions. With a photon at position (*i* + δ_1_, *j* + δ_2_) (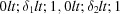), split the charges over four pixels: 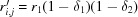, 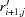 = 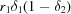, 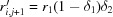, 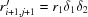. (iii) Use *r*_*i*,*j*_ to denote the readout of pixel (*i*, *j*). The error of the fitting is 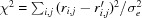. A least-squares fit is applied to minimize χ^2^ by iterating the photon positions. Step 3: repeat Step 2, fitting with new random starting positions until χ^2^ of the fitting is sufficiently small (χ^2^ < 0.5 with σ_e_ = 20) or until the maximum number of iterations (50 in our case) is reached.

*Psana Photon Converter*. Step 1: divide the pixel readout by *r*_1_ and split the readout into whole photons and fractional photons. Step 2: for the fractional photon map, search for a pixel that has at least 0.5 photons with an adjacent pixel that sum up to above 0.9 photons. Take the highest valued pixel if multiple pixels meet this requirement. Merge the two pixels values into the pixel with the higher value. Step 3: round the fractional photon map and combine with the whole photon map.

**Figure 3:**
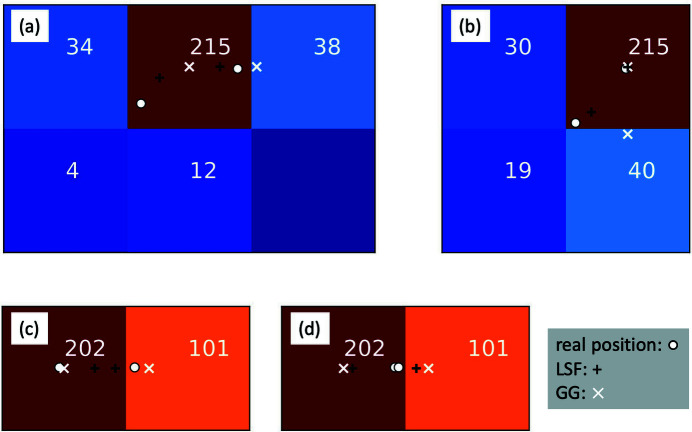
(*a*, *b*) Examples illustrating the two types of errors of photon assignment by the GG and PPC algorithms. (*c*, *d*) Photon position degeneracy. The numbers in white denote the pixel readout values assuming a single-photon ADU value of *r*_1_ = 151. Here all the examples are simulated assuming σ_r_ = 0 and σ_g_ = 0. The white circles, white ‘×’ and black ‘+’ are the real photon positions, and the estimated positions by GG and LSF separately.

Equation (1) indicates that the speckle contrast is mainly determined by obtaining an accurate measurement of the two photons per pixel events probability *P*(2) and the estimation of the count rate ∼*P*(1). For the GG algorithm, two primary factors can lead to its inaccuracy. First, as photons are assigned sequentially, subtracting the ADU equivalent to one photon or *r*_1_ from the pixel readout might be overestimating the contribution of the one photon that falls into that pixel but out of the 98% boundary. This leaves a high possibility that the residue readout will be less than 0.5*r*_1_ even if a second photon is present in this same pixel. One example of this is drawn in Fig. 3 ▸(*a*). This leads to an underestimate of *P*(2) and the contrast estimation. Second, when a photon hits the corner area enclosed by the solid black line, as in Fig. 2 ▸(*b*), the charges generated are shared by a 2 × 2 pixel droplet. As illustrated in Fig. 3 ▸(*b*), these ‘corner’ photons might not necessarily distribute charges more than 0.5*r*_1_ into the pixel where the photon arrived. In this case, instead of assigning a photon to this pixel, the GG algorithm will assign a photon to its brightest neighboring pixel. The two scenarios apply to the PPC algorithm as well, with an additional consequence that the second photon will not be counted because no pixel has a readout bigger than half a photon after substracting one photon from the brightest pixel. For the examples in Figs. 3 ▸(*a*) and 3(*b*), only one photon will be assigned. Both cases will result in a biased estimation of *P*(1) and *P*(2).

The errors of the LSF algorithm have different origins. It is more accurate as it fits to the readout values of all the pixels within each droplet rather than merely two adjacent pixels, as done in the GG and PPC algorithms. However, it has different bias during photon assignment due to two additional factors. First, in LSF Step 2 (ii), calculation of the pixel readout values is dependent on the assumptions of the charge cloud properties including shape and size. We can see the difference via the 50% charge contribution boundary shown in Fig. 2 ▸(*b*). Compared with the boundary calculated from the nominal parameters, it gives tighter space for a photon to supply at least half a photon charge to the pixel. This effectively clusters the photons towards the center of the pixels. Second, due to the finite detector pixel size and thus the down-sampling of the charges, there is a ‘degeneracy’ in the photon positions. Two configurations of photon positions can generate the same readout value for two-photon two-pixel droplets as shown in Figs. 3 ▸(*c*) and 3(*d*). The GG approach will always put one photon in each pixel, thus underestimating *P*(2) due to undercounting the Fig. 3 ▸(*d*) scenario. On the other hand, for the fitting method, moving the two photons’ fitting positions towards each other from Fig. 3(*d*) by the same amount gives the same *r*′_*i*,*j*_ calculated for fitting following LSF Step 2(ii). As a result, all these positions have the same χ^2^. Fitting this same droplet 10 000 times with random initialization gives us a 21% chance of getting two-photon events. While the occurrences of the photon positions of scenarios like Fig. 3(*d*) compared with Fig. 3(*c*) are less frequent. In order to quantify the impact, we randomly generated a large number of two-photon two-pixel droplets and used the LSF algorithm to estimate the photon positions. The occurrences of both photons being assigned to the same pixel was found to be overestimated by 10%.

**Figure 4:**
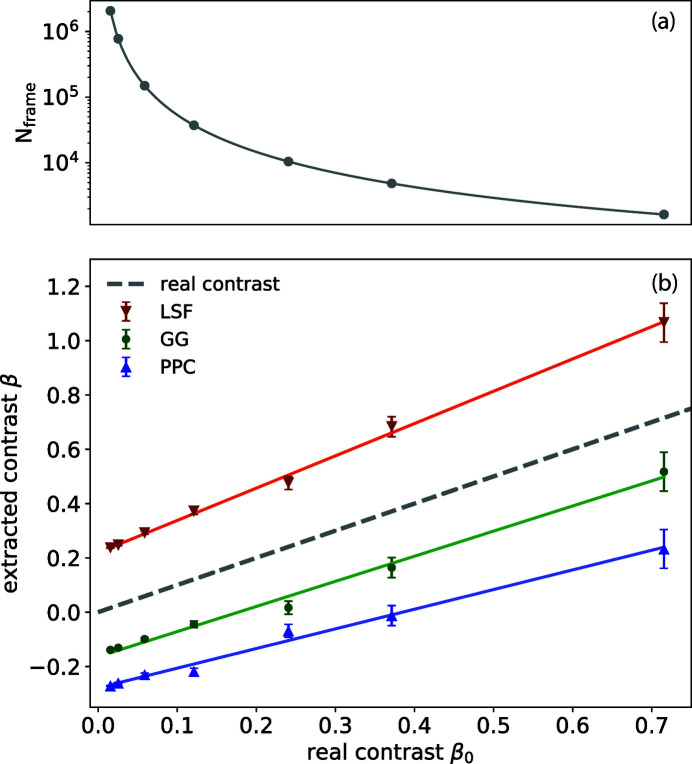
(*a*) Number of frames needed to achieve a 10% r.m.s. accuracy of contrast determination given *N*_pixel_ = 64 × 64. (*b*) Extracted contrast values from the three algorithms (LSF, GG and PPC) versus the real contrasts at a fixed count rate 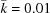 photons per pixel. The gray dashed line indicates the correct solution. The slopes for the three fitted lines for the extracted contrasts using LSF, GG and PPC are 1.2 ± 0.1, 0.93 ± 0.09 and 0.72 ± 0.07, respectively.

Shown in Fig. 4 ▸(*b*) are the extracted contrast values using the three different algorithms described above. All detector images are simulated with a uniform 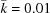 photons per pixel. The gray dashed line is the real contrast β_0_ we obtain by the definition of contrast,
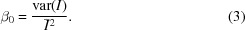
Here *I* is the intensity distribution of the speckle patterns used for simulating weak speckles. The extracted contrasts from all three algorithms show systematic deviations from the real contrasts. LSF overestimates the contrast while GG and PPC underestimate it. Yet all three methods show linear responses to changes in contrast. LSF has a fitted value for the slope that is greater than 1, while the slopes for the other two methods are less than 1. To explain this, we use Δ_*i*_ to denote the overestimate percentage of *i* photons per pixel events and derive its influence analytically. To the first order, Δ_*i*_ is related to the location where each photon hits the pixel and should have weak dependence on contrast. GG and LSF both include droplet identification as a first step and are thus more accurate with regards to the total number of photons or *P*(1), Δ_1_ ≃ 0. For the PPC algorithm, Δ_1_ < 0 as discussed earlier. Using equation (1), we estimate that the contrast β follows
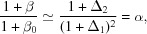
or
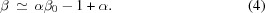
This indicates that the intercept and slope of the linear model are to the first order different by 1. The vertical offset at β_0_ = 0 can be determined experimentally by the *P*(1) and *P*(2) measurements of scattering signal with very low visibility such as when the scattering volume is enlarged to produce a very high level of undersampling of the speckle size.

## 4. Discussion on biased contrast   

In this section, we evaluate the influence of the three primary nonidealities of the detectors one by one. By varying the amplitude of one parameter while fixing the other two in our simulation of the detector images, we can understand how each parameter will influence the extracted contrast value.

### 4.1. Gain non-uniformity   

**Figure 5:**
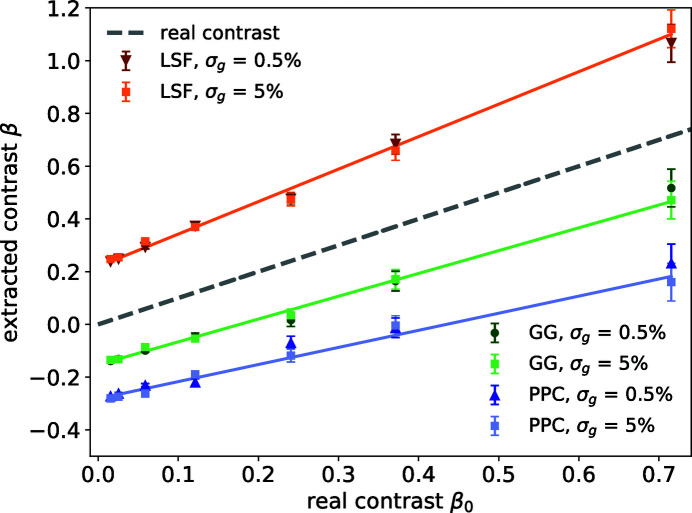
The extracted contrast values from different algorithms at fixed count rate 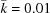 photons per pixel for both nominal gain variation σ_g_ = 0.5% and ten times larger σ_g_ = 5%. The gray dashed line indicates the correct solution. The three lines are fitted to extracted contrasts for σ_g_ = 5% using LSF, GG and PPC and their slopes are 1.2 ± 0.1, 0.87 ± 0.08 and 0.65 ± 0.07, respectively.

When the detector gain inhomogeneity σ_*g*_ is increased by a factor of ten from 0.5 to 5%, the detector pixel readout histogram changes significantly, as shown in Fig. 2 ▸(*a*) by the orange curve. However, when comparing the contrast extracted from these two scenarios, all three algorithms show very little sensitivity to this change. This is not surprising as the readout noise σ_r_ ≪ *r*_1_; we thus can set the boundaries for photon number identification wider than σ_*g*_, namely 50% of the nominal one-photon readout for a droplet for LSF and GG algorithms, and 10% for two neighboring pixels for PPC. As a result, the algorithms give consistent results even with a few percent variations of the pixel readout values due to their non-uniformity (see Fig. 5 ▸).

### 4.2. Readout noise   

**Figure 6:**
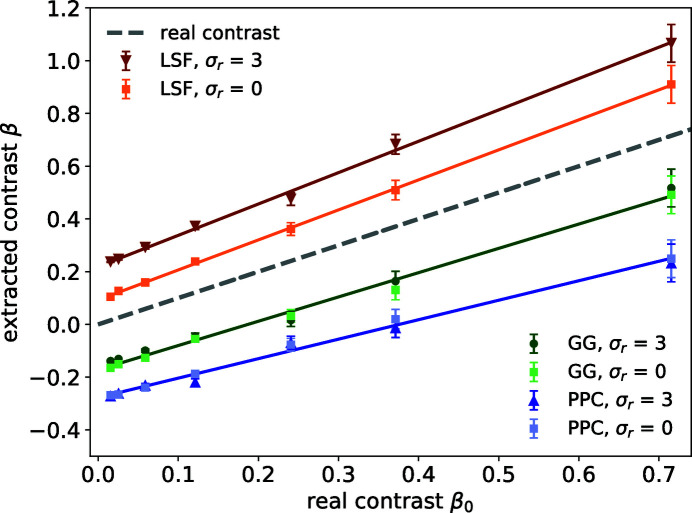
The extracted contrast values from different algorithms at fixed count rate 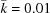 photons per pixel are for σ_*r*_ = 3 and σ_r_ = 0. For both cases, σ_g_ = 0.5% and *S* = 0.385. The gray dashed line indicates the correct solution. The slopes for the four fitted lines for the extracted contrast values using LSF with σ_r_ = 3, LSF with σ_r_ = 0, GG and PPC for both σ_*r*_ values are 1.2 ± 0.1, 1.1 ± 0.1, 0.93 ± 0.09 and 0.72 ± 0.07, respectively.

To understand the impact of detector readout noise, we start by considering a detector with zero electronic readout noise, *i.e.* σ_r_ = 0. The corresponding ADU histogram is plotted in Fig. 2 ▸(*a*) in green. In this case, we no longer need to threshold the detector readout values for the GG and LSF algorithms. The rest of the data processing stays the same. Plotted in Fig. 6 ▸ is the comparison between the noiseless detector case and our previous simulation with σ_r_ = 3 for the three algorithms. For GG and PPC, as they both have a tendency to assign photons to pixels with larger values [or to pixel (*i*, *j*) with *r*_*i*,*j*_ > 0.5*r*_1_ ≫ σ_r_], they show little dependence on σ_r_ when *r*_1_ ≫ σ_r_. This shows that a detector with lower readout noise is not expected to improve the accuracy of these two algorithms. On the other hand, for the LSF algorithm, a more accurate droplet readout distribution does improve the precision of the photon assignment. Moreover, when the detector readout noise is above 0, in the thresholding step with the boundary pixels peeled off, it effectively pushes the signal distribution towards the center of the droplets, thus has the tendency to overestimate the occurrences of multiple photons falling into the same pixel. This effect will be eliminated with a noiseless detector. However, the intrinsic degeneracy of pixel readout values is not expected to improve without a finer sampling of the charge cloud. As a result, in this case we see a fitted slope value of α ≃ 1.1 in Fig. 6 ▸. This corresponds to Δ_2_ ≃ 10%, which agrees with our prediction of the overestimate percentage due to the degeneracy in the two-photon two-pixel droplets discussed earlier.

### 4.3. Charge cloud size   

**Figure 7:**
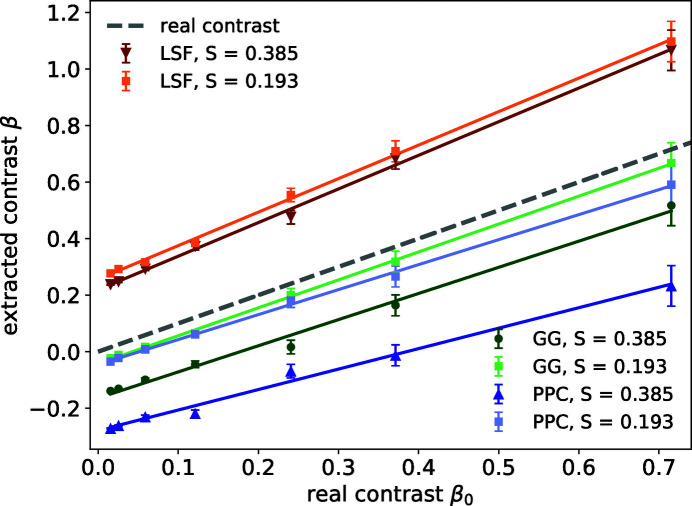
The extracted contrast values from different algorithms at fixed count rate 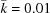 photons per pixel for both nominal charge cloud size *S* = 0.385 and half that size *S* = 0.193. The gray dashed line indicates the correction solution. The three fitted lines for *S* = 0.193 using LSF (orange), GG (light green) and PPC (light purple) give slope values of 1.2 ± 0.1, 0.99 ± 0.1 and 0.88 ± 0.09, respectively.

Finally, we look at the impact of the charge cloud size to the contrast evaluation. As the charge cloud shrinks, the probability of the droplets taking the same forms as in Figs. 3 ▸(*a*) and 3(*b*) decreases due to the expansion of the 98% and 50% area and the reduction of the corner area as plotted in Fig. 2 ▸(*c*). This explains why the extracted contrasts by GG and PPC both get close to the real contrast as we reduce the charge cloud size by 50% as shown in Fig. 7 ▸. For LSF, even though the probability of the ‘degeneracy’ becomes lower, the placement of photons in LSF Step 2(ii) deviates from the exact fit even more. This is because for smaller charge cloud sizes, the charge cloud is less well measured by the pixel grid and thus the fitting becomes less precise. Moreover, the comparison of the 50% charge contribution boundary plotted in Figs. 2 ▸(*b*) and 2(*c*) suggests that the LSF algorithm has a larger tendency to cluster the photons to the center of the pixel. As a result, the LSF algorithm still overestimates the number of two-photon events.

## 5. Contrast extraction considering pulse energy fluctuations   

The discussions so far have been conducted in the context of having a stable average count rate, *i.e.* 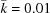 photons per pixel. In a real-life XPCS experiment, the X-ray FEL pulses originating from the self-amplified spontaneous emission process are often further reduced in bandwidth by a monochromator. The intensities of the output pulses on the sample follows the Gamma distribution (Sun *et al.*, 2019 ▸). Maximum-likelihood estimation was formulated to provide an unbiased fit considering frames with a non-uniform count rate (Roseker *et al.*, 2018 ▸). One of the prerequisites is that the algorithm for extracting photon probabilities has no dependency on scattering intensities. However, Δ_2_ can depend on the count rate as it changes the distribution of droplets: the average droplet size is expected to grow as the photon density increases. The various photon assignment algorithms can have a different bias for droplets of different sizes and shapes.

**Figure 8:**
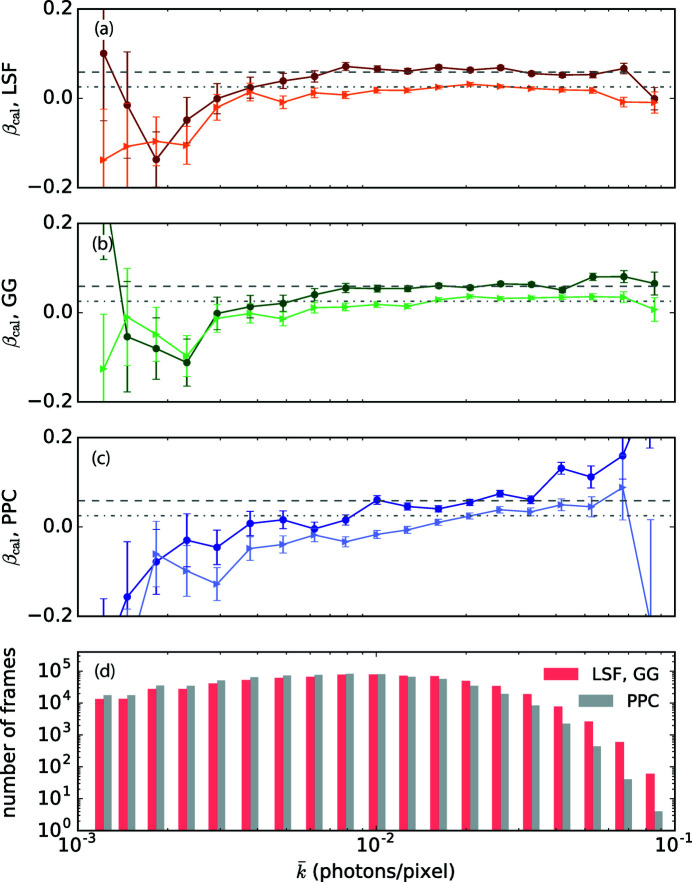
Calibrated contrast values as a function of mean photon count rate 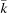 obtained from (*a*) LSF in orange, (*b*) GG in green and (*c*) PPC in purple for two datasets with known different contrast levels. The two known contrast levels are plotted in gray dashed lines. (*d*) Histogram of the number of simulated detector frames falling into each 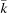 bin for the three algorithms.

We recreated datasets that mimic the real experimental conditions assuming an incoming pulse energy distribution that corresponds to a mean mode number of 1.3 (Gutt *et al.*, 2009 ▸), which also produces an average scattering intensity 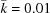 photons per pixel on the detector. Speckle patterns with two slightly different contrast values are generated (0.06 and 0.03). Each simulated dataset contains 0.8 million frames of 64 × 64 pixel speckle patterns. We bin our data based on 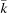 determined by the three algorithms and then use the linear fit in Fig. 4 ▸ to correct the contrast evaluation. The calibrated contrasts of LSF, GG and PPC are plotted in Figs. 8 ▸(*a*)–8(*c*) as a function of 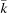. In the count rate regime with sufficient statistics, all three methods show sensitivity to distinguish between the two datasets. The calibrated contrasts at the same contrast but with different 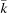 deviate from a straight horizontal line, indicating an intensity dependent systematic error. The LSF and GG algorithms have a precise determination of photon numbers for each frame, while the PPC algorithm has a biased estimation on the number of photons. This is evident in the deviation of their histograms of the mean count rate as displayed in Fig. 8 ▸(*d*). This also explains why PPC is most influenced by the average count rate in its contrast evaluation. The finding here is that one should always compare the extracted contrast values at the same average detector intensity level experimentally to assert that the observed contrast change originates from dynamics rather than the systematic errors of contrast extraction algorithms.

## 6. Conclusions   

In conclusion, using numerical simulation of detector response, we were able to identify a set of parameters to closely mimic the behavior of real hard X-ray detectors. Using these parameters, we simulated a large number of low-count-rate speckles and performed error analysis of the commonly used photon locating algorithms. The three algorithms examined all have systematic errors but all show a consistent linear response to contrast changes with similar signal-to-noise performance. This suggests that calibration using scattering with known contrast, especially the 0 contrast point, is essential for measurement of a ‘normalized’ or correct contrast value, otherwise data interpretation can be significantly biased. The simple algorithms which can be easily run on-the-fly, *i.e.* Greedy Guess and Psana Photon Converter, will enable live feedback in the experiment (see Appendix *B* [App appb]). They are also much easier to implement at the hardware level for next generation MHz Mega-pixel sensors to produce processed information with highly reduced size, rather than the raw data. We further compare the systematic errors (deviation of extracted contrast from real contrast) by varying the parameter values of detector ‘nonidealities’. We found that the charge cloud size has the largest impact, yet, for all three algorithms, this did not severely impact the sensitivity to contrast change measurements. Finally, when the pulse-to-pulse energy fluctuations are taken into consideration, our analysis suggests that algorithms based on droplets show more consistency, probably due to their accurate determination of the total number of photons.

## Figures and Tables

**Table 1:** Computation time consumed for the three algorithms to extract photon positions from the 800 detector images (*N*_pixel_ = 64 × 64) at count rates of 0.1, 0.01 and 0.001 photons per pixel using a single core of Intel(R) Xeon(R) CPU E5-2620 v4 processor. The error bars following the computation time indicate the range of time fluctuations in the ten program executions

Time (s)	0.1 (photons pixel^−1^)	0.01 (photons pixel^−1^)	0.001 (photons pixel^−1^)
PPC	0.059 (0.003)	0.059 (0.003)	0.058 (0.003)
GG	2.6 (0.1)	0.125 (0.003)	0.057 (0.003)
LSF	2.8 (0.1) × 10^3^	2.5 (0.1)	0.065 (0.003)

## Appendix A Photon statistics for contrast error estimation

The probabilities of multiple photons per pixel events follow a negative binomial distribution (Goodman, 2007 ▸),

Here *M* is related to the measured speckle visibility with β = 1/*M*. At the sparse photon limit or , the Taylor expansion of the probabilities for *k* = 1 and *k* = 2 are

and

From this, we can derive an estimation of visibility,

For a region of interest containing *N*_pixel_ pixels on the detector, the number of frames needed (*N*_frame_) in order to determine the contrast β with a r.m.s. error δβ can be calculated by the derivative of *P*(2),

and considering the signal-to-noise ratio of photon counting

We therefore have

## Appendix B Computation time

Displayed in Table 1 ▸ is the computation time comparison between the three algorithms for three different average photon count rates. We note here that as the photon assignment process is highly parallelizable, all three algorithms can be easily scaled to provide live feedback. Nevertheless, simple algorithms such as PPC and GG are much more computationally economical, and potentially more realistic to deploy at the detector hardware/firmware level.
